# Interpretable machine learning models for early prediction of acute kidney injury after cardiac surgery

**DOI:** 10.1186/s12882-023-03324-w

**Published:** 2023-11-07

**Authors:** Jicheng Jiang, Xinyun Liu, Zhaoyun Cheng, Qianjin Liu, Wenlu Xing

**Affiliations:** 1 Department of Big Data Center for Cardiovascular Disease, Heart Center of Henan Provincial People’s Hospital, Fuwai Central China Cardiovascular Hospital, Central China Fuwai Hospital of Zhengzhou University, Zhengzhou, China; 2 Department of Cardiovascular Surgery, Heart Center of Henan Provincial People’s Hospital, Fuwai Central China Cardiovascular Hospital, Central China Fuwai Hospital of Zhengzhou University, Zhengzhou, China

**Keywords:** Acute kidney Injury, Cardiac surgery, Machine learning, Predictive model

## Abstract

**Objective:**

Postoperative acute kidney injury (PO-AKI) is a common complication after cardiac surgery. We aimed to evaluate whether machine learning algorithms could significantly improve the risk prediction of PO-AKI.

**Methods:**

The retrospective cohort study included 2310 adult patients undergoing cardiac surgery in a tertiary teaching hospital, China. Postoperative AKI and severe AKI were identified by the modified KDIGO definition. The sample was randomly divided into a derivation set and a validation set based on a ratio of 4:1. Exploiting conventional logistic regression (LR) and five ML algorithms including decision tree, random forest, gradient boosting classifier (GBC), Gaussian Naive Bayes and multilayer perceptron, we developed and validated the prediction models of PO-AKI. We implemented the interpretation of models using SHapley Additive exPlanation (SHAP) analysis.

**Results:**

Postoperative AKI and severe AKI occurred in 1020 (44.2%) and 286 (12.4%) patients, respectively. Compared with the five ML models, LR model for PO-AKI exhibited the largest AUC (0.812, 95%*CI*: 0.756, 0.860, all *P* < 0.05), sensitivity (0.774, 95%*CI*: 0.719, 0.813), accuracy (0.753, 95%*CI*: 0.719, 0.781) and Youden index (0.513, 95%*CI*: 0.451, 0.573). Regarding severe AKI, GBC algorithm showed a significantly higher AUC than the other four ML models (all *P* < 0.05). Although no significant difference (*P* = 0.173) was observed in AUCs between GBC (0.86, 95%*CI*: 0.808, 0.902) and conventional logistic regression (0.803, 95%*CI*: 0.746, 0.852), GBC achieved greater sensitivity, accuracy and Youden index than conventional LR. Notably, SHAP analyses showed that preoperative serum creatinine, hyperlipidemia, lipid-lowering agents and assisted ventilation time were consistently among the top five important predictors for both postoperative AKI and severe AKI.

**Conclusion:**

Logistic regression and GBC algorithm demonstrated moderate to good discrimination and superior performance in predicting PO-AKI and severe AKI, respectively. Interpretation of the models identified the key contributors to the predictions, which could potentially inform clinical interventions.

**Supplementary Information:**

The online version contains supplementary material available at 10.1186/s12882-023-03324-w.

## Introduction

Postoperative acute kidney injury (PO-AKI) is a common complication after cardiac and vascular surgery that is strongly associated with increased risk of in-hospital mortality, prolonged intensive care unit (ICU) admission and hospital stay, and greater healthcare costs [1]. Its prevalence has been reported to range from 20 to 70% and vary by the type of surgery and the criteria of the AKI definition [2, 3]. Even minor increases in serum creatinine after cardiac surgery matter and add to the risk of death [4]. Despite the fact that many episodes of postoperative AKI are reversible within days or weeks of onset, results from a number of epidemiological studies conducted over the last decade reveal a substantial association of AKI with subsequent chronic kidney disease and long-term adverse consequences [5, 6], which leads to a considerable disease burden. The pathophysiological mechanisms of PO-AKI are multifactorial and still largely unknown. Currently, the health care of PO-AKI patients is mainly based on supportive therapy. The best approach to AKI management is prevention. The success of many preventative treatment strategies rests on the ability to correctly identify patients at high risk of developing AKI prior to major changes in serum creatinine or urine output. Therefore, perioperative accurate assessment of AKI risk and specific preventive strategies are urgent to be established. To early identify patients at higher risk for PO-AKI, previous studies have developed a number of potentially useful clinical risk prediction models. But the generalization of the models is uncertain. Most models suffer from differing AKI definitions, insufficient perioperative variables, small cohorts, and a lack of external validation [7]. Moreover, these models were designed mainly for western populations and did not represent good performance among Chinese patients.

In recent years, machine learning (ML) techniques have also been increasingly employed to make predictions and have shown promise as a potentially powerful tool to detect data patterns and trends that could remain undiscovered using traditional statistical method [8–12]. Decision trees (DT) are versatile and intuitive models that use a tree-like structure to make decisions or predictions. Random Forest (RF) is an ensemble learning method that combines multiple decision trees to make predictions by aggregating the votes or averaging the outputs of individual trees. Gradient Boosting Classifier (GBC) is another ensemble learning method that builds a predictive model by combining multiple weak classifiers in a stage-wise manner. Gaussian Naive Bayes (GNB) is a probabilistic classification algorithm based on Bayes’ theorem and calculates the posterior probability of each class for a given instance and assigns the class with the highest probability as the predicted class. Multilayer Perceptron (MLP) is a type of artificial neural network that consists of multiple layers of interconnected nodes. These algorithms have their own strengths and weaknesses. It’s important to experiment and evaluate different algorithms to find the best fit for a specific task. Compared with the CSM approach, ML algorithms could theoretically perform better in measuring nonlinear associations, dealing with high-dimensional data, which may enhance risk prediction. However, the existing studies did not systematically compare and statistically test the difference in performance among ML algorithms and the CSM approach in predicting PO-AKI [13]. Some of the existing ML models did not report more interpretable indicators for model assessment, such as sensitivity and specificity, which were highly emphasized in clinical conditions [8, 14].

Interpretation of ML models is another crucial issue that refers to the challenge of understanding how and why a machine learning model arrived at a particular prediction or decision. Machine learning models are often regarded as black boxes due to their complex and nonlinear nature. This lack of transparency hinders the understanding of the factors driving predictions and can limit their adoption in critical applications. However, several approaches can be used to enhance model interpretability and overcome this limitation. Techniques such as SHAP (SHapley Additive exPlanations) method or permutation feature importance assign weights or rankings to each feature based on their impact on the model’s output, thereby enabling insights into the underlying factors and increasing the trustworthiness of predictive models. Although previous studies suggested that ML approaches provided good prediction discrimination, the interpretability of the models was lacking, which limited their application in an actual clinical setting.

Therefore, we hypothesized that ML techniques may outperform traditional logistic regression analysis in the prediction of postoperative AKI risk following cardiac surgery. The study aimed to systematically compare the performance of multiple different ML algorithms and LR in predicting two outcomes separately, including postoperative AKI and severe AKI as well. We also sought to further explain the prediction of the best-performing model at the population and individual levels.

## Methods

### Study design

In the present retrospective cohort study, we enrolled 2327 patients who were over eighteen years old and underwent cardiac surgeries in a tertiary teaching hospital during January 2018 through December 2020. A total of 17 patients were excluded according to the criteria below: (a) preoperative dialysis or renal transplantation (4 cases); (b) used nephrotoxic drugs within seven days before surgery: gentamicin and vancomycin (2 cases); (c) preoperative or postoperative serum creatinine was not available (11 cases). The rationale behind the exclusion was to avoid the influence of the baseline factors above on postoperative renal function or PO-AKI diagnosis. Finally, 2310 participants were eligible for the present analysis. The study was approved by the Medical Ethics Committee of Central China Fuwai Hospital of Zhengzhou University (LUNSHEN 2020-07). This work has followed the TRIPOD (transparent reporting of a multivariable prediction model for individual prognosis or diagnosis) guidance for the reporting of studies developing and validating a prediction model [15].

### Data collection

We applied to the China Cardiac Surgery Registry (CCSR) database for the data submitted by our institute. CCSR was a web-based and paperless data collection system. Data quality control measures for the CCSR database were described in another study [16] introducing the design and data audit of the CCSR. Further, to ensure the accuracy and reliability of the data, two investigators screened the data for the missing or outlier values and replaced them with the true values extracted from electronic health records (EHR). The available variables regarding demographics, comorbidities, previous cardiac interventions, preoperative medications, perioperative examinations, intraoperative data, postoperative complications and outcomes were included in the present analyses. Based on evidence from previous studies, clinical guidelines, or expert opinions, predictor selection during model development was conducted among preoperative and intraoperative variables.

Demographics and presentation of comorbidities included gender, age, BMI, smoker regardless of whether the patient quit smoking, diabetes mellitus, hypertension, hyperlipidemia, chronic renal failure, chronic obstructive pulmonary disease (COPD), peripheral vascular disease, cerebrovascular accident (CA), congestive cardiac failure, Canadian Cardiovascular Society (CCS) angina class, New York Heart Association (NYHA) class, arrhythmia, and previous myocardial infarction (MI). Previous cardiac intervention included previous percutaneous coronary intervention (PCI) and previous cardiac surgery. Preoperative medications included intravenous nitroglycerin injection, catecholamine injection, β-blockers, angiotensin-converting-enzyme inhibitor (ACEi)/ angiotensin II receptor blocker (ARB), lipid-lowering agents. Perioperative examinations included the last preoperative serum creatinine (SCr), peak postoperative SCr, last preoperative left ventricular ejection fraction (LVEF), last preoperative left ventricular end-diastolic diameter (LVEDD), number of diseased coronary vessels. Intraoperative data included surgery type, cardiopulmonary bypass (CPB), aortic cross-clamping (ACC), intra-aortic balloon pump (IABP) usage, intraoperative blood product transfusion (BPT), postoperative BPT, and assisted ventilation time. BPT refers to packed red blood cells (pRBC), fresh frozen plasma, cryoprecipitate and platelet.

### Outcomes

We considered AKI and severe AKI after cardiac surgery as the primary and secondary outcomes, respectively. AKI diagnosis was based on a modified KDIGO (Kidney Disease: Improving Global Outcomes) definition [17] by using serum creatinine (SCr) criteria with the most recent preoperative result as baseline. AKI was defined as any of the following: SCr increase ≥ 0.3 mg/dL within 48 h or SCr increase ≥ 1.5 times baseline within 7 days of surgery. Severe AKI (stage 2 and 3 AKI classification) refers to the condition of an increase in SCr ≥ 4.0 mg/dL or ≥ 2.0 times baseline or the initiation of renal replacement therapy (RRT).

### Statistical analysis

During data preprocessing, the nonphysiologic vital values or outliers were checked and replaced by the true values from electronic medical records when it was mis-recorded or changed to missing. For several variables with missing data (BMI, CCS angina class, COPD), we imputed the missing values by random forest - multiple imputation method in R using the ‘missForest’ package.

For all participants grouped by postoperative AKI or severe AKI, the continuous variables were presented as mean ± standard deviation (SD) and analyzed by unpaired t tests or expressed as median (interquartile range) and compared using the Mann-Whitney U test when they violated the normality assumption. The discrete variables were expressed as frequencies (%) and tested using Chi-square tests.

### Model development, validation and comparisons

For model development, the complete dataset was randomly divided into a derivation set and a validation set based on a ratio of 4:1. The derivation set was used to train the model and the validation set was used to access the generalizability of the model.

To reduce redundant data dimensions, univariate analyses were performed to select feature predictors. Only the candidate preoperative and intraoperative variables that were significantly associated with PO-AKI would be incorporated in the model training.

In the derivation dataset, we used traditional logistic regression analysis and five popular supervised ML algorithms to train models, including decision tree (DT), random forest (RF), gradient boosting classifier (GBC), Gaussian Naive Bayes (GNB) and multilayer perceptron (MLP).

Furthermore, the predictive value of each algorithm in discriminating PO-AKI was systematically evaluated in the validation set by using sensitivity, specificity, area under the receiver operating characteristic curve (AUC), accuracy, Youden index (sensitivity plus specificity-1) and the 95% confidence interval (*CI*). Sensitivity measures the ability of a model to correctly identify positive cases out of all the actual positive cases. Specificity measures the ability of a model to correctly identify negative cases out of all the actual negative cases. AUC is a commonly used metric that summarizes the overall performance of a model to discriminate between positive and negative instances across various classification thresholds. Accuracy calculates the proportion of correctly classified instances over the total number of instances. Youden index is derived from sensitivity and specificity and provides a balance between these two metrics, allowing for the determination of an optimal classification threshold. The statistical significance of the difference among the AUCs was further examined using the method proposed by DeLong et al. (1988) using MedCalc® Statistical Software version 20. This method is a widely used approach for comparing AUC values between two or more models. It is based on the nonparametric approach and uses the theory of generalized U-statistics.

### Model interpretation using SHAP

Lastly, we focused on interpreting the output of the final ML models. SHAP (SHapley Additive exPlanations) is a unified approach [18] to explain the variable impact on the output of any model by assigning each feature consistent and accurate attribution values for a particular prediction. The benefits of utilizing SHAP are as follows: (1) the collective SHAP values help to interpret and understand the model at a global level, (2) an observation gets its own set of SHAP values that explain the variable impact on individual predictions at a local level. We further visualized the SHAP explanation by summary plot and force plot to understand how the models interpret the data and make predictions.

Statistical analyses were conducted with SAS version 9.1 (SAS Institute Inc., Cary, North Carolina, USA). Development, validation and interpretation of the models were performed under Python Version 3.6 environment. All P values were two-sided with a significance level of 0.05.

## Results

### Comparison of characteristics and outcomes

A total of 2310 participants were included in the present retrospective cohort study. Table 1 shows the comparison of characteristics and outcomes in patients with and without postoperative AKI or severe AKI. In the whole cohort of cardiac surgery, 1020 (44.2%) patients developed PO-AKI and 286 (12.4%) developed severe AKI (stage 2 and 3 AKI). Univariate analyses indicated that presentation of hyperlipidemia, cerebrovascular accident, congestive cardiac failure, CCS angina class II-IV, NYHA class III-IV, CABG + valve/other surgery, CPB, aortic cross-clamping, IABP usage, intraoperative BPT, and longer assisted ventilation time were significantly associated with increased risk of PO-AKI (all *P* < 0.05). Notably, we found that the levels of preoperative SCr in the patients without PO-AKI were significantly higher than those of patients with PO-AKI (*P* < 0.001). Compared to the patients with PO-AKI, those without PO-AKI had higher percentages of preoperative medications use, such as intravenous nitroglycerin injection (54.1% vs. 43.3%; *P* < 0.001), β-blockers (50.1% vs. 37.8%; *P* < 0.001), ACEi/ARB (17.8% vs. 10.4%; *P* < 0.001), lipid-lowering agents (41.1% vs. 21.0%; *P* < 0.001)). There was no statistical significance between AKI and no-AKI groups regarding gender, age, BMI, smoker, diabetes mellitus, hypertension, chronic renal failure, COPD, peripheral vascular disease, arrhythmia, previous MI and cardiac interventions.

**Table 1:** Characteristics of the patients with and without postoperative AKI/severe AKI

	No AKI (n = 1290, 55.8%)	AKI (n = 1020, 44.2%)	*P*	No severe AKI (n = 2024, 87.6%)	Severe AKI (n = 286, 12.4%)	*P*
Male	922 (71.5%)	736 (72.2%)	0.72	1482 (73.2%)	176 (61.5%)	< 0.001
Age (years)	61.00 (54.00, 66.00)	61.00 (55.00, 66.00)	0.86	60.50 (54.00, 65.00)	63.00 (56.00, 68.00)	< 0.001
BMI (kg/m^2^)	25.14 (23.14, 27.33)	25.64 (23.44, 27.68)	0.06	25.29 (23.23, 27.49)	25.68 (23.70, 27.68)	0.076
Smoker	540 (41.9%)	422 (41.4%)	0.81	854 (42.2%)	108 (37.8%)	0.15
Diabetes mellitus	348 (27.0%)	250 (24.5%)	0.18	528 (26.1%)	70 (24.5%)	0.56
Hypertension	648(50.2%)	546 (53.5%)	0.12	1048(51.8%)	146 (51.0%)	0.80
Hyperlipidemia	594 (46.0%)	536 (52.5%)	0.002	970 (47.9%)	160(55.9%)	0.011
Chronic renal failure	6 (0.5%)	10 (1.0%)	0.14	16 (0.8%)	0 (0.0%)	0.13
COPD	4 (0.3%)	2 (0.2%)	0.59	6 (0.3%)	0 (0.0%)	0.36
Peripheral vascular disease	8 (0.6%)	8 (0.8%)	0.64	16 (0.8%)	0 (0.0%)	0.13
Cerebrovascular accident	128 (9.9%)	144 (14.1%)	0.002	228 (11.3%)	44 (15.4%)	0.04
Congestive cardiac failure	68 (5.3%)	82 (8.0%)	0.01	128 (6.3%)	22 (7.7%)	0.38
CCS angina class II-IV	1112 (86.2%)	934 (91.6%)	< 0.001	1776 (87.7%)	270 (94.4%)	< 0.001
NYHA class III-IV	904 (70.1%)	838 (82.2%)	< 0.001	1488 (73.5%)	254 (88.8%)	< 0.001
Arrhythmia	70 (5.4%)	50(4.9%)	0.57	110 (5.4%)	10 (3.5%)	0.17
Previous MI	354 (27.4%)	322 (31.6%)	0.05	572 (28.3%)	104 (36.4%)	0.005
Previous PCI	46 (3.6%)	42 (4.1%)	0.49	80 (4.0%)	8 (2.8%)	0.34
Previous cardiac surgery	6 (0.5%)	4 (0.4%)	0.79	8 (0.4%)	2 (0.7%)	0.46
Intravenous nitroglycerin injection^#1^	698 (54.1%)	442 (43.3%)	< 0.001	1036 (51.2%)	104 (36.4%)	< 0.001
Catecholamine injection^#2^	20 (1.6%)	10 (1.0%)	0.24	24 (1.2%)	6 (2.1%)	0.20
β-blockers^#1^	646 (50.1%)	386 (37.8%)	< 0.001	936 (46.2%)	96 (33.6%)	< 0.001
ACEi/ARB^#2^	230 (17.8%)	106 (10.4%)	< 0.001	308 (15.2%)	28 (9.8%)	0.015
Lipid-lowering agents^#1^	530 (41.1%)	214 (21.0%)	< 0.001	700 (34.6%)	44 (15.4%)	< 0.001
Last preoperative Scr (mg/dl)	0.78 (0.67, 0.91)	0.73 (0.58, 0.88)	< 0.001	0.77 (0.66, 0.90)	0.65 (0.51, 0.82)	< 0.001
Last preoperative LVEF (%)	59.00 (55.00, 62.00)	59.00 (55.00, 62.00)	0.087	59.00 (55.00, 62.00)	59.00 (54.00, 62.00)	0.002
Last preoperative LVEDD (mm)	50.00 (47.00, 55.00)	51.00 (48.00, 56.00)	0.099	51.00 (48.00, 55.00)	51.00 (48.00, 55.00)	0.39
Number of diseased coronary vessels	3.00 (3.00, 3.00)	3.00 (3.00, 3.00)	0.16	3.00 (3.00, 3.00)	3.00 (3.00, 3.00)	0.90
CABG + valve/other surgery	80 (6.2%)	100 (9.8%)	0.001	146 (7.2%)	34 (11.9%)	0.01
Elective surgery	1282 (99.4%)	1014 (99.4%)	0.82	2012 (99.4%)	284 (99.3%)	0.36
CPB	298 (23.1%)	422 (41.4%)	< 0.001	594 (29.3%)	126 (44.1%)	< 0.001
Aortic cross-clamping	226 (17.5%)	260 (25.5%)	< 0.001	408 (20.2%)	78 (27.3%)	0.01
IABP	52 (4.0%)	100 (9.8%)	< 0.001	100 (4.9%)	52 (18.2%)	< 0.001
Intraoperative BPT	346 (26.8%)	392 (38.4%)	< 0.001	612 (30.2%)	126 (44.1%)	< 0.001
Postoperative BPT	1100 (85.3%)	868 (85.1%)	0.91	1732 (85.6%)	236 (82.5%)	0.17
Assisted ventilation time (hr)	18.50 (15.00, 24.00)	21.00 (16.00, 36.00)	< 0.001	19.00 (15.50, 25.00)	21.50 (16.50, 49.50)	< 0.001

^#1^ within 24 h before operation; ^#2^ within 48 h before operation

Abbreviations: AKI, acute kidney injury; BMI, body mass index; COPD, chronic obstructive pulmonary disease; CCS, Canadian Cardiovascular Society; NYHA, New York heart association; MI, myocardial infarction; PCI, previous percutaneous coronary intervention; ACEi, angiotensin-converting-enzyme inhibitor; ARB, angiotensin II receptor blocker; SCr, serum creatinine; LVEF, left ventricular ejection fractions; LVEDD, left ventricular end-diastolic diameter; CABG, coronary artery bypass grafting; CPB, cardiopulmonary bypass; IABP, intra-aortic balloon pump; BPT, blood product transfusion

### Development, validation and comparison of models

**Fig. 1:**
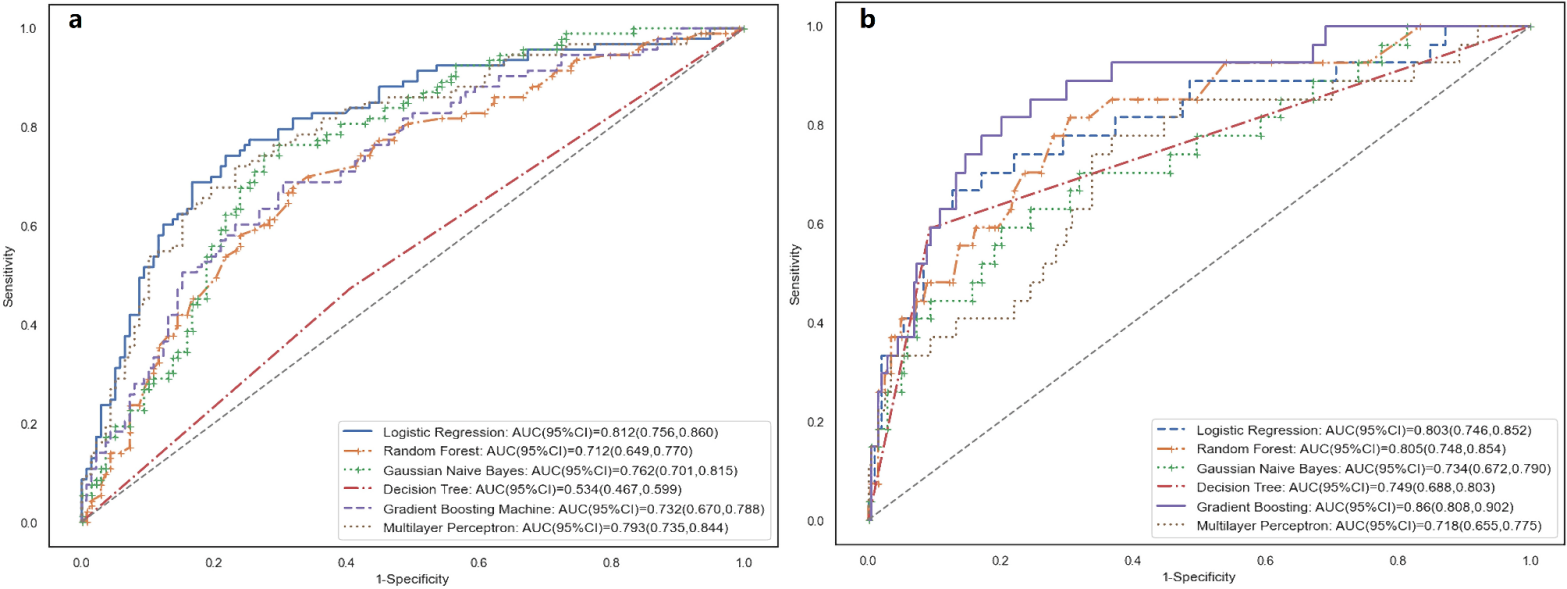
Model performance illustrated by receiver operating characteristic curves for predicting (**a**) postoperative AKI and (**b**) postoperative severe AKI. AKI, acute kidney injury; AUC, area under receiver operating characteristic curves; CI, confidence interval

Using the sixteen preoperative and intraoperative predictors that were significantly associated with postoperative AKI, we developed five ML models and a conventional logistic regression model in the derivation set (n = 1848, 80%) and validated the models in the validation set (n = 462, 20%) for prediction of postoperative AKI and severe AKI, respectively. There were no significant differences among patient characteristics, postoperative outcome and complications between the derivation set and the validated set (Supplementary Table 1). Model performance metrics in the validated set were demonstrated in Table 2. The ROC curves of prediction models were also illustrated in Fig. 1(a) for AKI prediction and Fig. 1(b) for severe AKI prediction. Comparisons of AUCs among different models are presented in Supplementary Table 2.

**Table 2:** Performance metrics of the models for prediction of postoperative AKI/ Severe AKI in the deviation set

Outcome	Model	Sensitivity (95%CI)	Specificity (95%CI)	AUC^#^ (95%CI)	Acurracy (95%CI)	Youden index (95%CI)
AKI	LR	0.774(0.719,0.813)	0.739(0.698,0.784)	0.812(0.756,0.860)	0.753(0.719,0.781)	0.513(0.451,0.573)
	DT	0.473(0.420,0.537)	0.594(0.544,0.635)	0.534(0.467,0.599)	0.545(0.516,0.570)	0.067(-0.010,0.194)
	RF	0.602(0.552,0.657)	0.725(0.673,0.777)	0.712(0.649,0.770)	0.675(0.626,0.709)	0.327(0.238,0.379)
	GBC	0.581(0.525,0.629)	0.775(0.725,0.814)	0.732(0.670,0.788)	0.697(0.672,0.742)	0.356(0.294,0.436)
	GNB	0.452(0.358,0.507)	0.826(0.781,0.859)	0.762(0.701,0.815)	0.675(0.644,0.702)	0.278(0.205,0.347)
	MLP	0.656(0.575,0.729)	0.804(0.749,0.843)	0.793(0.735,0.844)	0.745(0.705,0.778)	0.460(0.391,0.536)
Severe AKI	LR	0.148(0.078,0.250)	0.990(0.982,0.997)	0.803(0.746,0.852)	0.892(0.877,0.905)	0.138(0.065,0.260)
	DT	0.593(0.483,0.707)	0.907(0.882,0.930)	0.749(0.688,0.803)	0.870(0.841,0.894)	0.500(0.393,0.596)
	RF	0.296(0.181,0.388)	0.975(0.964,0.985)	0.805(0.748,0.854)	0.896(0.875,0.912)	0.271(0.147,0.348)
	GBC	0.333(0.224,0.431)	0.971(0.955,0.983)	0.86(0.808,0.902)	0.896(0.868,0.917)	0.304(0.202,0.424)
	GNB	0.407(0.284,0.517)	0.922(0.894,0.942)	0.734(0.672,0.790)	0.861(0.835,0.886)	0.329(0.219,0.428)
	MLP	0.074(0.009,0.138)	1.000(1.000,1.000)	0.718(0.655,0.775)	0.892(0.888,0.898)	0.074(0.026,0.147)

Abbreviations: AKI, acute kidney injury; AUC, area under receiver operating characteristic curves; CI, confidence interval; LR, Logistic Regression; DT, Decision Tree; RF, Random Forest; GBC, Gradient Boosting Classifier; GNB, Gaussian Naive Bayes; MLP, Multilayer Perceptron;

Among the five ML models, MLP and GNB, with similar AUCs (0.793, 95%*CI*: 0.735, 0.844 vs. 0.762, 95%*CI*: 0.701, 0.815, *P*_difference_ =0.179), performed best in predicting postoperative AKI. Compared to MLP, logistic regression had a higher AUC (0.812, 95%*CI*: 0.756, 0.860 vs. 0.793, 95%*CI*: 0.735, 0.844, *P*_difference_ =0.036), sensitivity (0.774, 95%*CI*: 0.719, 0.813 vs. 0.804, 95%*CI*: 0.749, 0.843), accuracy (0.753, 95%*CI*: 0.719, 0.781 vs. 0.745, 95%*CI*: 0.705, 0.778) and Youden index (0.513, 95%*CI*: 0.451, 0.573 vs. 0.460, 95%*CI*: 0.391, 0.536).

For postoperative severe AKI prediction, in terms of AUC, GBC (0.86, 95%*CI*: 0.808, 0.902) performed significantly better than the other four ML models, including DT (0.749, 95%*CI*: 0.688, 0.803, *P*_difference_ =0.011), RF (0.805, 95%*CI*: 0.748, 0.854, *P*_difference_ =0.027), GNB (0.734, 95%*CI*: 0.672, 0.790, *P*_difference_ =0.004) and MLP (0.718, 95%*CI*: 0.655, 0.775, *P*_difference_ <0.001). The AUC of GBC was larger than the AUC of conventional logistic regression (0.86, 95%*CI*: 0.808, 0.902 vs. 0.803, 95%*CI*: 0.746, 0.852) but no significant differences were found (*P*_difference_ =0.173). As illustrated in Table 2, the sensitivity (0.333, 95%CI: 0.224, 0.431 vs. 0.148, 95%CI: 0.078, 0.250), Youden index (0.304, 95%*CI*: 0.202, 0.424 vs. 0.138, 95%*CI*: 0.065, 0.260) and accuracy (0.896, 95%*CI*: 0.868, 0.917 vs. 0.892, 95%*CI*: 0.877, 0.905) of GBC seemed to be consistently greater than those of conventional logistic regression.

### Model interpretation

**Fig. 2:**
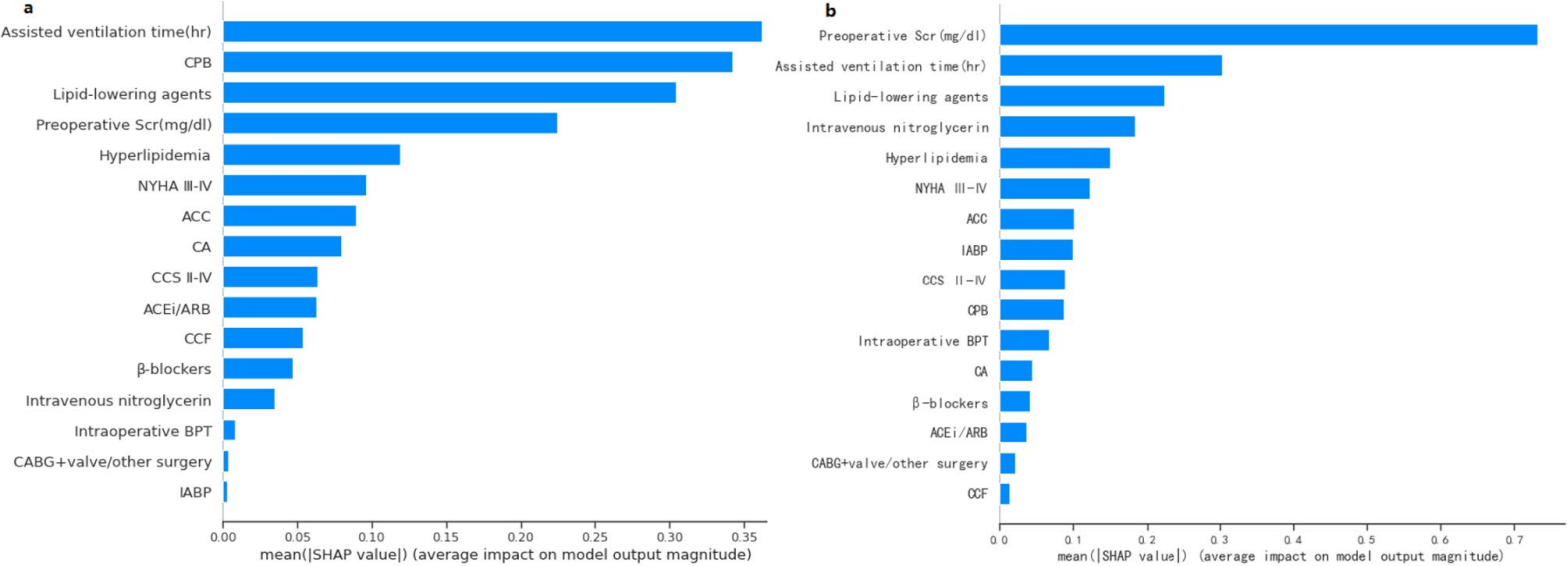
Feature importance matrix plot based on SHAP for (**a**) postoperative AKI prediction using logistic regression and (**b**) postoperative severe AKI prediction using gradient boosting model. AKI, acute kidney injury; CCS, Canadian Cardiovascular Society; NYHA, New York heart association; CA, cerebrovascular accident; ACEi, angiotensin-converting-enzyme inhibitor; ARB, angiotensin II receptor blocker; ACC, Aortic cross-clamping; SCr, serum creatinine; CABG, coronary artery bypass grafting; CPB, cardiopulmonary bypass; IABP, intra-aortic balloon pump; BPT, blood product transfusion

**Fig. 3:**
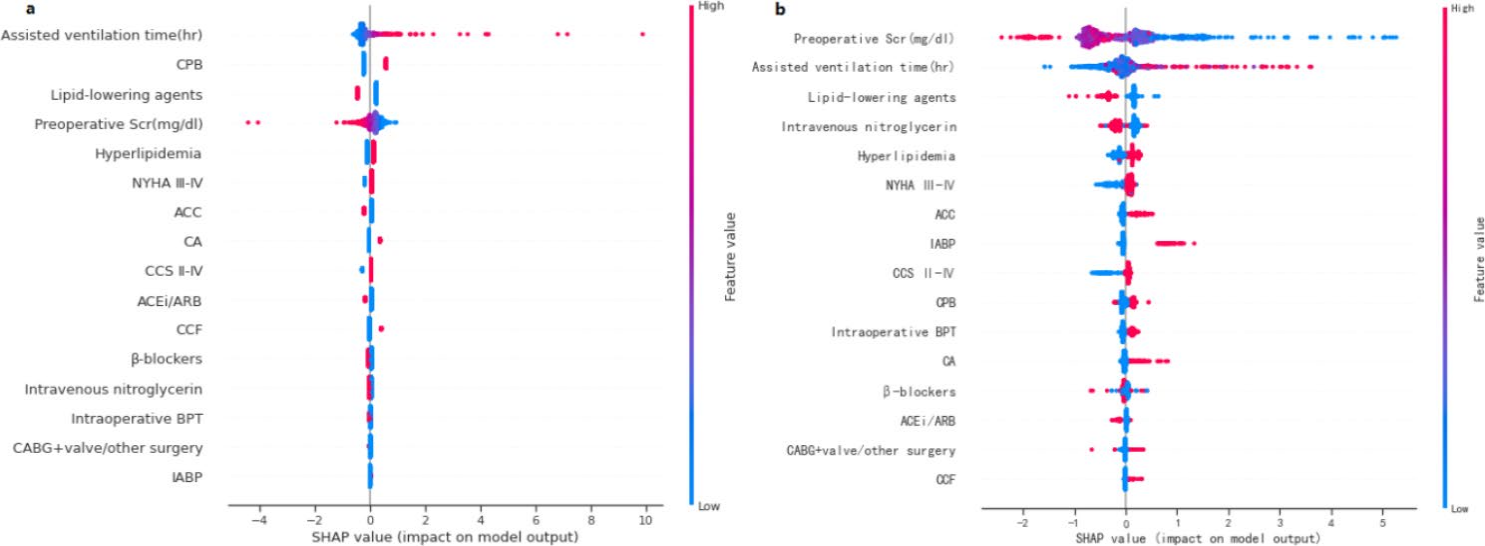
SHAP summary plot for (**a**) postoperative AKI prediction using logistic regression and (**b**) postoperative severe AKI prediction using gradient boosting model. AKI, acute kidney injury; CCS, Canadian Cardiovascular Society; NYHA, New York heart association; CA, cerebrovascular accident; ACEi, angiotensin-converting-enzyme inhibitor; ARB, angiotensin II receptor blocker; ACC, Aortic cross-clamping; SCr, serum creatinine; CABG, coronary artery bypass grafting; CPB, cardiopulmonary bypass; IABP, intra-aortic balloon pump; BPT, blood product transfusion

For AKI prediction, the feature importance matrix plot for the best-performing logistic regression is shown in Fig. 2 (a). The top five most important predictors based on the SHAP values were assisted ventilation time, CPB, lipid-lowering agents, last preoperative SCr, and hyperlipidemia. To summarize the effects of all the features on logistic regression output at a global level, we plotted the SHAP values of every feature for every participant (Fig. 3 (a)). It revealed that CPB and longer assisted ventilation time increased the risk of AKI and vice versa for preoperative lipid-lowering agents and higher preoperative SCr. At a local level, we could get a set of SHAP values for specific participant’s feature values and visualize the impact of each feature on the output by force plot (Supplementary Fig. 1(a)). The color of the arrows shows how the feature impacts the model: a red arrow increases the model output predicted value in the positive direction while a blue arrow decreases it in the negative direction. The size of the color arrows for each feature value represents the impact magnitude of the SHAP value. The base value refers to the mean probability that would be predicted if we do not know any features for the current output. As shown in Supplementary Fig. 1(a), the base value here was 0.49 and the output predicted value was 0.61, lipid-lowering agents = 0 with red arrow and ACC = 1 with the blue arrow have similar impact magnitudes but in opposite directions. Red feature values contribute to postoperative AKI prediction, while blue ones push towards no AKI development.

Similarly, we combined the optimal GBC model for severe AKI prediction with SHAP analysis and presented the importance matrix plot in Fig. 2 (b). To get an overview of which features contribute most to the GBC model, we used the SHAP summary plot (Fig. 3 (b)). Of the top five most important variables, there were two risk factors (longer assisted ventilation time and hyperlipidemia) and three protective factors (higher last preoperative SCr, preoperative lipid-lowering agents and intravenous nitroglycerin intake). SHAP force plot (Supplementary Fig. 1(b)) showed the impact direction and magnitude of each feature value on the development of postoperative severe AKI by taking a specific patient case as an example. In this case, the blue-colored explanatory variable values (last preoperative SCr (mg/dl) = 0.49, no preoperative intravenous nitroglycerin injection, no preoperative intake of lipid-lowering agents, NYHA III-IV, no preoperative intake of β-blockers, hyperlipidemia) had a positive contribution towards severe AKI development and outweighed the negative impact of other blue-colored variable values, such as no CPB, assisted ventilation time (hr) = 45 and so on.

## Discussions

We conducted a retrospective study to investigate the incidence and risk factors of patients developing PO-AKI and compare the performance of machine learning algorithms and logistic regression in predicting PO-AKI following cardiac surgery. Contrary to our expectations, traditional logistic regression outperformed all five other machine learning models in discriminating postoperative AKI. However, Gradient Boosting Classifier showed higher AUC values than traditional logistic regression when predicting postoperative severe AKI, but no significant difference was found. Taking into account sensitivity, accuracy, and Youden index, GBC appeared to be superior to traditional logistic regression in predicting postoperative severe AKI. The study yielded moderate to good predictive values for both postoperative PO-AKI and severe AKI. Furthermore, the models identified various risk factors, including lower preoperative SCr level, longer assisted ventilation time, cardiopulmonary bypass and hyperlipidemia, and discovered main protective factors, such as preoperative lipid-lowering agents and intravenous nitroglycerin intake, which could potentially inform clinical interventions.

The performance of risk prediction model was typically determined by various factors, such as the quality of the data, sample size, predictor parameters, algorithms and so on. Lee et al. [9] reported that XGboost achieved the best performance (AUC: 0.78 (95%*CI* 0.75–0.80)) using 39 variables to predict postoperative AKI of all stages compared to traditional logistic regression (AUC: 0.69 (95%*CI* 0.66–0.72)) and other ML models. On the contrary, based on the same AKI definition and similar incidence of postoperative AKI, we used far fewer predictors (16 variables) and found that the performance of traditional logistic regression (AUC: 0.812(95%*CI* 0.756–0.860)) was significantly superior to that of other five ML models in predicting PO-AKI of all stages. Notably, we have also attempted to include as many variables as possible in the models before formal analyses. However, we found that the expansion of input features did not imply great improvement of predictive ability, probably due to high correlation between the features or data noise. Thottakkara et al. [19] found that reducing the dimensionality of the predictors with LASSO had minimal effect on the performance of models, while feature extraction using principal component analysis improved predictive performance. We were convinced that models with redundant data and elaborate risk calculation would add extra burden and make it difficult to deploy in real clinical conditions. So we only included the variables significantly associated with postoperative AKI, which may have the risk of omitting some potentially important variables but aid in decreasing overfitting and making the model easier interpretable. Additionally, more advanced and complex algorithms are not certainly synonymous with substantial performance improvement. For example, Tomašev et al. [20] once utilized a broad range of alternative RNN architectures (specifically, the long short-term memory, update gate RNN and intersection RNN, the neural Turing machine, memory-augmented neural network, simple recurrent units, gated recurrent units, the Differentiable Neural Computer and the relational memory core) to create a continuous prediction model of AKI. These alternatives did not show significantly incremental performance than the simple recurrent unit architecture.

Notably, the performance of risk prediction models should be assessed comprehensively and compared based on scientific statistical tests. To identify more positive cases and reduce false negative cases, sensitivity is particularly emphasized over specificity in clinical screening and risk assessment. Excellent discrimination measured by AUC does not necessarily mean good performance in sensitivity. However, some previous studies [3, 8, 9, 21] did not report such more comprehensive metrics for model assessment, which may overestimate their models’ reliability and practical values. Additionally, we could not rule out the possibility of publication bias. Studies that demonstrate better performance with ML algorithms may be more likely to be published compared to those showing insignificant results. This can impact the overall body of evidence by potentially skewing the performance of ML models.

Even if logistic regression outperformed the ML models in discrimination of postoperative AKI, the limitations of logistic regression should be addressed as well. Logistic regression assumes a linear relationship between the independent variables and the logit function. It may not capture non-linear relationships and complex interactions among variables. Logistic regression has limitations in handling high-dimensional data, missing data and unbalanced datasets. Logistic regression relies on manual or statistical methods for feature selection, which requires prior knowledge or assumptions about the relevant variables. Anyway, we could not underestimate the strengths of ML algorithms. Bihorac et al. [22] used an automated generalized additive model (GAM) with logistic link function to assess the risk of death and eight postoperative complications including AKI and reported an AUC of 0.88 (0.87–0.89) and a sensitivity of 0.80 (0.78, 0.82) for AKI after index surgery. The advantages of this automated ML system included automatic covariate selection, universal applicability to any type of surgery, exportability to other EHR systems, and the ability to handle high-dimensional datasets with multiple different data types (such as interrelated, correlated, semistructured or unstructured data, missing or sparse data). Rank et al. [23] built a recurrent neural network (RNN) based on 96 routinely collected clinical parameters for real-time prediction of AKI within 8 future time horizons (6-h, 12-h, 18-h, 24-h, 36-h, 48-h, 60-h and 72-h ahead) after cardiothoracic surgery. The deep-learning model yielded a significantly higher AUC and sensitivity than the experienced clinicians. The RNN model could potentially be integrated into an EHR for real-time patient monitoring and early detection of postoperative AKI. A shift to personalized predictive medicine, where large amounts of multiple different types of features are integrated, suggests that computational methods like ML would become increasingly popular.

While model performance is crucial, the interpretability and clinical implications of the model also play significant roles. As revealed by SHAP analysis, LR and GBC models have identified lower preoperative SCr level as an important contributor to the development of PO-AKI, particularly in cases of postoperative severe AKI. Intriguingly, patients who experienced severe AKI exhibited significantly lower preoperative SCr level (0.65 (95% *CI* 0.51, 0.82) mg/dl vs. 0.77 (95% *CI* 0.66, 0.90) mg/dl) and higher postoperative SCr level (1.57 (95% *CI* 1.24, 2.31) mg/dl vs. 0.98 (95% *CI* 0.82, 1.16) mg/dl) than patients without severe AKI. Conversely, Teresa et al. [24] and Guan et al. [25] found that patients with AKI following cardiac surgery had higher baseline SCr level. Renal functional reserve (RFR) may help explain the opposite phenomenon we observed. RFR is defined by the difference between maximal functional capacity and baseline function and represents the capacity of the kidneys to increase GFR in response to stimuli (e.g., protein load or during pregnancy) [26, 27]. Those who appear to have sustained no permanent loss of kidney function (normal serum creatinine), may still lose RFR. In the present study, patients with lower baseline SCr level may have higher baseline function and diminished RFR, which makes the renal response to cardiac surgery less powerful and induced kidney injury. Ronco et al. [28] have demonstrated that preoperative RFR, measured with a protocolized protein load of 1.2 g/kg, was highly predictive of cardiac surgery associated-AKI with an AUC of 0.83 (95% *CI* 0.70–0.96) and patients with an increased renal reserve were less likely to develop postoperative AKI. Husain-Syed et al. [29] noted that patients with postoperative AKI have a persistent decrease in RFR at 3 months after cardiac surgery despite normalization of serum creatinine and patients with severe AKI exhibited higher decreases in RFR. These remind us that preoperative detection of baseline SCr is not enough and highlight the importance of assessing renal functional reserve as a highly potential predictor of postoperative AKI before cardiac surgery. The patients with normal baseline SCr but occult impaired renal reserve should be timely identified and deserve close clinical attention.

The models also established hyperlipidemia and preoperative lipid-lowering agents use as among the top five important predictors for postoperative AKI and severe AKI as well. In this study, statins accounted for over 97% of the lipid-lowering agents used preoperatively. It is speculated that preoperative lipid-lowering agents (e.g., statins) use may be protective against AKI by inhibiting post-operative inflammatory processes. Several randomized controlled trials investigating inflammatory markers and perioperative statin usage in patients undergoing cardiac surgery have demonstrated a reduction in inflammatory cytokines [30]. Meta-analyses of observational studies have revealed that preoperative statin therapy significantly reduced the incidence of postoperative renal dysfunction and the requirement for renal replacement therapy in patients undergoing cardiac surgery [31, 32]. Similarly, other modifiable factors such as assisted ventilation time, cardiopulmonary bypass and intravenous nitroglycerin intake were also found to be key predictors of AKI risk, highlighting their clinical implications in predicting AKI and guiding clinical intervention.

Some advantages of the study could be acknowledged. We comprehensively developed, validated and compared the traditional logistic regression and the five most popular ML models in the prediction of postoperative AKI and severe AKI as well. More variables on preoperative medicine intakes and intraoperative procedures were included as modifiable predictors, which provide potential targets for prevention and intervention of high-risk population. Furthermore, we made intuitive explanations of the models using SHAP at the global and local levels, which shed light on the black box of ML and aid in understanding how and how much each predictor contributes. To determine whether sufficient power was gained to ensure the reliability of our results, we implemented a post hoc sample size calculation using the method proposed by Riley et al. [33] via the pmsampsize package in R. The number of predictor parameters was 16, the R^2^ was 0.11, and the overall AKI incidence was 44.2%. Finally, the minimum sample size with 33.92 events per predictor required for model development based on the inputs is 1228. The actual sample of 1848 patients for model development likely has sufficient power to draw conclusions.

### Limitation

However, the study also presented several limitations. First, the diagnosis of AKI was based on the modified KDIGO definition without incorporating urine output criteria. Because the collection and measurement of urine are sometimes incomplete, inaccurate and unreliable. In addition to the impact of renal function, urine output varies depending on the use of diuretic or fluid treatment regimens. Second, due to the unavailability of the complete time-series data, the study could not make real-time risk prediction of AKI. Future enhancement would include integrating additional perioperative time-series data into the prediction models. Third, the models needed to be further externally validated. Factors such as differences in demographic characteristics, comorbidities, surgical profiles, management protocols, and perioperative care practices may influence the performance of the models. Moreover, the impact of potential confounding factors, such as variations in baseline kidney function and medication regimens, should also be carefully considered when applying these models in different populations or settings. Therefore, rigorous external validation studies are necessary to verify the performance and establish the clinical utility of these prediction models in diverse patient populations and healthcare settings.

## Conclusions

The study did not provide consistent evidence supporting the hypothesis that machine learning algorithms could significantly enhance the predictive performance of postoperative AKI and severe AKI after cardiac surgery compared to conventional logistic regression. Logistic regression and the GBC algorithm showed superior performance and exhibited moderate to good predictive ability for the risk of PO-AKI and severe AKI, respectively. We interpreted the models at the population and individual level, which gained insight into why those predictions were made and offered evidence for individual prevention and treatment.

### Electronic supplementary material

Below is the link to the electronic supplementary material.

Supplementary Material 1

## Data Availability

Data are available from the corresponding author on reasonable request.
