# Supplementary material for: Interpretable machine learning models for early prediction of acute kidney injury after cardiac surgery
Source: BMC Nephrol. 2023 Nov 7;24:326. doi: 10.1186/s12882-023-03324-w (PMC10631004; doi:10.1186/s12882-023-03324-w)
Supplement: Supplementary file 1 — Supplementary Material 1 [file 12882_2023_3324_MOESM1_ESM.docx]

**Legends for the supplementary**

Supplementary Table 1. Distribution of patient characteristics in the training set and test set.

Supplementary Table 2. Comparisons of predictive performance between different models for prediction of postoperative AKI/ Severe AKI.

Supplementary Figure 1. Details of how each feature contributes to the individual prediction of (a) postoperative AKI using logistic regression and (b) postoperative severe AKI using gradient boosting model presented by SHAP force plot.

Supplementary Table 1. Distribution of patient characteristics, postoperative outcome and complications in the derivation set and validation set.

|  | Derivation set  (n=924, 80%) | Validation set  (n=231, 20%) | P |
| --- | --- | --- | --- |
| Male | 673 (72.8%) | 156 (67.5%) | 0.11 |
| Age(years) | 61.00 (55.00, 66.00) | 61.00 (54.00, 66.00) | 0.55 |
| BMI(kg/m2) | 25.30 (23.33, 27.43) | 25.69 (23.18, 27.82) | 0.28 |
| Smoker | 392 (42.4%) | 89 (38.5%) | 0.28 |
| Diabetes mellitus | 245 (26.5%) | 54 (23.4%) | 0.33 |
| Hypertension | 472 (51.1%) | 125 (54.1%) | 0.41 |
| Hyperlipidemia | 454 (49.1%) | 111 (48.1%) | 0.77 |
| Chronic renal failure | 5 (0.5%) | 3 (1.3%) | 0.21 |
| COPD | 3 (0.3%) | 0 (0.0%) | 0.39 |
| Peripheral vascular disease | 8 (0.9%) | 0 (0.0%) | 0.16 |
| Cerebrovascular accident | 107 (11.6%) | 29 (12.6%) | 0.68 |
| Congestive cardiac failure | 58 (6.3%) | 17 (7.4%) | 0.55 |
| CCS angina class Ⅱ-Ⅳ | 816 (88.3%) | 207 (89.6%) | 0.58 |
| NYHA class Ⅲ-Ⅳ | 698 (75.5%) | 173 (74.9%) | 0.84 |
| Arrhythmia | 51 (5.5%) | 9 (3.9%) | 0.32 |
| Atrial fibrillation | 38 (4.1%) | 9 (3.9%) | 0.88 |
| Previous MI | 268 (29.0%) | 70 (30.3%) | 0.70 |
| Previous PCI | 35 (3.8%) | 9 (3.9%) | 0.94 |
| Previous cardiac surgery | 4 (0.4%) | 1 (0.4%) | 1.00 |
| Intravenous nitroglycerin injection^#1^ | 455 (49.2%) | 115 (49.8%) | 0.88 |
| catecholamine injection^#2^ | 13 (1.4%) | 2 (0.9%) | 0.51 |
| β-blockers^#1^ | 413 (44.7%) | 103 (44.6%) | 0.98 |
| ACEi/ARB^#2^ | 138 (14.9%) | 30 (13.0%) | 0.45 |
| Lipid-lowering agents^#1^ | 299 (32.4%) | 73 (31.6%) | 0.83 |
| Aspirin^#3^ | 30 (3.2%) | 6 (2.6%) | 0.61 |
| Clopidogrel ^#3^ | 28 (3.0%) | 6 (2.6%) | 0.73 |
| Last preoperative Scr(mg/dl) | 0.75 (0.64, 0.89) | 0.77 (0.65, 0.92) | 0.30 |
| Last preoperative LVEF(%) | 59.00 (55.00, 62.00) | 59.00 (55.00, 62.00) | 0.31 |
| Last preoperative LVEDD(mm) | 51.00 (48.00, 55.00) | 51.00 (48.00, 56.00) | 0.55 |
| Number of diseased coronary vessels | 3.00 (3.00, 3.00) | 3.00 (3.00, 3.00) | 0.20 |
| CABG+valve/other surgery | 72 (7.8%) | 18 (7.8%) | 1.00 |
| Elective surgery | 917 (99.2%) | 231 (100.0%) | 0.41 |
| CPB | 285 (30.8%) | 75 (32.5%) | 0.63 |
| Aortic cross-clamping | 194 (21.0%) | 49 (21.2%) | 0.94 |
| IABP | 63 (6.8%) | 13 (5.6%) | 0.51 |
| Intraoperative BPT | 287 (31.1%) | 82 (35.5%) | 0.20 |
| Postoperative BPT | 787 (85.2%) | 197 (85.3%) | 0.97 |
| Assisted ventilation time(hr) | 19.50 (16.00, 26.00) | 19.50 (16.00, 26.00) | 0.42 |
| Peak postoperative Scr(mg/dl) | 89.00 (74.00, 109.00) | 90.00 (74.00, 115.00) | 0.63 |
| Stroke | 1 (0.1%) | 0 (0.0%) | 0.62 |
| Renal failure | 9 (1.0%) | 0 (0.0%) | 0.13 |
| Atrial fibrillation | 8 (0.9%) | 0 (0.0%) | 0.16 |
| Death | 9 (1.0%) | 1 (0.4%) | 0.43 |
| Hospital stay(days) | 24.00 (20.00, 32.00) | 26.00 (21.00, 32.00) | 0.059 |
| AKI | 417 (45.1%) | 93 (40.3%) | 0.18 |
| Severe AKI | 112 (12.1%) | 31 (13.4%) | 0.59 |

^#1^ within 24 hours before operation; ^#2^ within 48 hours before operation; ^#3^ within 5 days before operation;

Abbreviations: AKI, acute kidney injury; CI, confidence interval; BMI, body mass index; COPD, chronic obstructive pulmonary disease; CCS, Canadian Cardiovascular Society; NYHA, New York heart association; MI, myocardial infarction; PCI, previous percutaneous coronary intervention; ACEi, angiotensin-converting-enzyme inhibitor; ARB, angiotensin II receptor blocker; SCr, serum creatinine; LVEF, left ventricular ejection fractions; LVEDD, left ventricular end-diastolic diameter; CABG, coronary artery bypass grafting; CPB, cardiopulmonary bypass; IABP, intra-aortic balloon pump; BPT, blood product transfusion; pRBC, packed red blood cell.

Supplementary Table 2. Comparisons of predictive performance between different models for prediction of postoperative AKI/ Severe AKI

| Comparision of models | AKI | |  | Severe AKI | |
| --- | --- | --- | --- | --- | --- |
|  | Difference between AUCs  (95%CI) | *P* |  | Difference between AUCs  (95%CI) | *P* |
| Decisiontree ~ GaussianNB | 0.228(0.146,0.310) | <0.001 |  | 0.014(-0.094,0.123) | 0.796 |
| Decisiontree ~ GradientBoosting | 0.198(0.126,0.271) | <0.001 |  | 0.111(0.026,0.196) | 0.011 |
| Decisiontree ~ LogisticRegression | 0.279(0.203,0.354) | <0.001 |  | 0.054(-0.051,0.160) | 0.312 |
| Decisiontree ~ MLPerceptron | 0.26(0.184,0.335) | <0.001 |  | 0.031(-0.073,0.135) | 0.563 |
| Decisiontree ~ RandomForest | 0.179(0.111,0.246) | <0.001 |  | 0.056(-0.032,0.144) | 0.210 |
| GaussianNB ~ GradientBoosting | 0.030(-0.028,0.088) | 0.311 |  | 0.125(0.040,0.211) | 0.004 |
| GaussianNB ~ LogisticRegression | 0.051(0.009,0.092) | 0.017 |  | 0.069(-0.020,0.157) | 0.127 |
| GaussianNB ~ MLPerceptron | 0.032(-0.015,0.078) | 0.179 |  | 0.016(-0.089,0.121) | 0.761 |
| GaussianNB ~ RandomForest | 0.049(-0.015,0.114) | 0.136 |  | 0.071(-0.015,0.156) | 0.105 |
| GradientBoosting ~ LogisticRegression | 0.081(0.037,0.124) | <0.001 |  | 0.057(-0.025,0.139) | 0.173 |
| GradientBoosting ~ MLPerceptron | 0.062(0.020,0.104) | 0.004 |  | 0.142(0.064,0.220) | <0.001 |
| GradientBoosting ~ RandomForest | 0.019(-0.023,0.062) | 0.373 |  | 0.055(0.006,0.103) | 0.027 |
| LogisticRegression ~ MLPerceptron | 0.019(0.0012,0.036) | 0.036 |  | 0.085(-0.0193,0.189) | 0.110 |
| LogisticRegression ~ RandomForest | 0.100(0.045,0.155) | <0.001 |  | 0.002(-0.069,0.073) | 0.954 |
| MLPerceptron ~ RandomForest | 0.081(0.027,0.135) | 0.003 |  | 0.087(0.018,0.156) | 0.013 |

Abbreviations: AKI, acute kidney injury; CI, confidence interval;


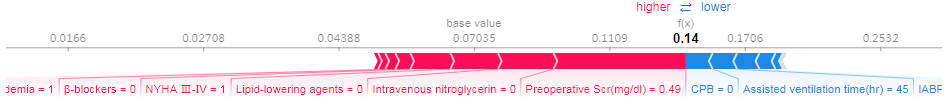

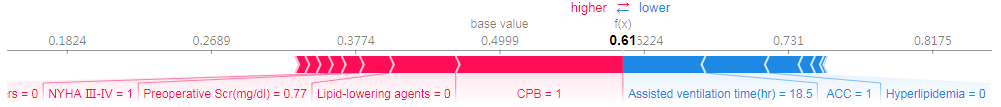


Supplementary Figure 1. Details of how each feature contributes to the individual prediction of (a) postoperative AKI using logistic regression and (b) postoperative severe AKI using gradient boosting model presented by SHAP force plot. AKI, acute kidney injury; CCS, Canadian Cardiovascular Society; NYHA, New York heart association; CA, cerebrovascular accident; ACEi, angiotensin-converting-enzyme inhibitor; ARB, angiotensin II receptor blocker; ACC, Aortic cross-clamping; SCr, serum creatinine; CABG, coronary artery bypass grafting; CPB, cardiopulmonary bypass; IABP, intra-aortic balloon pump; BPT, blood product transfusion.

**a**

**b**
